# Villari Effect at Low Strain in Magnetoactive Materials

**DOI:** 10.3390/ma13112472

**Published:** 2020-05-29

**Authors:** Graciela Riesgo, Laura Elbaile, Javier Carrizo, Rosario Díaz Crespo, María Ángeles García, Yadir Torres, José Ángel García

**Affiliations:** 1 Centro de Seguridad Marítima Integral Jovellanos, Camín del Centro de Salvamento n 279, 33393 Gijón, Spain; gracielarg@centrojovellanos.es; 2 Departamento de Física, Universidad de Oviedo, c/Calvo Sotelo s/n, 33007 Oviedo, Spain; elbaile@uniovi.es (L.E.); carrizo@uniovi.es (J.C.); charo@uniovi.es (R.D.C.); joseagd@uniovi.es (J.Á.G.); 3 Departamento de Ciencia de los Materiales, Universidad de Oviedo, c/Independencia n 13, 33004 Oviedo, Spain; magarc@uniovi.es; 4 Departamento de Ingeniería y Ciencia de los Materiales y el Transporte, Universidad de Sevilla, 41011 Sevilla, Spain

**Keywords:** magnetostrictive composites, Villari effect, magnetoactive materials, soft magnetic material

## Abstract

Magnetic composites of soft magnetic FeGa particles embedded in a silicone matrix have been synthesized. The Villari effect has been studied depending on the size and concentration of the particles and on the magnetic state of the composite. The results indicate a decrease in the Villari effect when the concentration of the magnetic particles increases. These results suggest a relationship between the Villari effect and the mechanical properties of the composites. The Young’s modulus of the composites has been obtained by microindentation and their values related to the intensity and slope of the Villari signals. The results are explained on the basis that the reduction in the cross section of the composite when submitted to stress is the main origin of the variation of the magnetic flux in the Villari effect in this kind of composite. It has also been obtained that the magnetic state of the composite plays an important role in the Villari signal. When the magnetization of the composite is greater, the magnetic flux across the composite is greater too and, so, the same reduction in the cross section originates a greater Villari signal.

## 1. Introduction

Magnetoactive composites are smart materials whose properties depend on the applied magnetic field. In these materials, the mechanical properties of an elastomer matrix are combined with the magnetic properties of the filler particles and so, the type, concentration and alignment of the filler particles affect the properties of the composite. Due to the dipole-dipole interaction of the magnetic particles, the application of an external magnetic field produces an increase in the Young’s modulus of the material which is known as magnetorheological effect. In this way, a material with a magnetically tunable Young’s modulus is obtained. These materials have high magnetostriction which depends on the percentage of ferromagnetic material [1] and the arrangement of the particles in the matrix. For isotropic magnetorheological elastomers, an extension along the applied magnetic field was found [2], while a compression was obtained in anisotropic ones [3].

These composites used to be made with iron particles of nano or micro sizes dispersed in a silicon polymer matrix [4,5,6,7]; however, recently other matrices: polymers, polyester, rubbers, thermoplastic elastomers, etc. and particles: ferrites (of barium, cobalt, iron and strontium), alloys of FeAl, and FaGa, etc., are being used [8,9,10,11,12].

Firstly, these materials were developed and studied only as magnetorheological elastomers due to the dependence of their mechanical properties on the applied magnetic field [13,14]. In this sense, nowadays there is intense research in composites formed by an elastomer matrix and hard magnetic materials [15,16,17]. These materials have properties similar to those made with materials with soft magnetic properties, but without the need to apply a magnetic field. In addition, when the hard magnetic particles are aligned during the manufacture process a flexible magnet is obtained.

Nowadays, the magnetoactive materials are applied in different technical applications such as magnetic dumping [18], actuators [19], in the vehicle industry as dynamic vibration absorbers [20], and sensing [21]. These materials have also been used in different biomedical applications: in peristaltic devices like micropumps [22], in drug delivery to control the time and amount of drug delivery from a reservoir [23], in hyperthermia due to the possibility of inducting heat in a magnetic elastomer by an alternating magnetic field [24] and to produce artificial muscles due to their mechanical properties and the frequency at which the strain drops to half of this amplitude [25].

The large magnetostriction in these materials makes them of great interest for use in actuators and sensors [26,27]. In recent years, the energy harvesting is emerging as an alternative to conventional batteries. The conversion of mechanical energy into electrical energy is obtained by the Villari effect [28,29]. This effect is one of the many magnetomechanical effects [30], and it is related directly to the change of the magnetic permeability of the material due to the magnetic anisotropy induced by stress. There is a great deal of literature on this subject in the ’classical’ bulk soft magnetic materials, such as amorphous and ferrites [31,32,33,34]. However, although the magnetostriction in composites is well studied [7,35], there are few works about the Villari effect [36,37,38,39]. In a monolithic material, the magnetostriction and the Villari effect are closely linked because the Villari effect is due only to a magnetic effect; however, in magneto elastomers materials there is another contribution, due to the mechanical properties of the matrix [40,41].

In addition, some studies theoretical [42] and experimental [43] related to the effect of the application of uniform magnetic fields on the behavior of ferrogels are reported in the literature. It was found that the spatial distribution of the magnetic particles plays an essential role in the magnetodeformation effect. In the work carried out by Zubarev et al. [42], indicated that an increase in the particle concentration in the composite can qualitatively change the type of magnetodeformation. For its part, in the work of Safronov et al. [44], the behavior of magnetic materials to manufacture biosensors used in biomedical applications is studied. The development of ferrogels (FG) with micron sized magnetic particles of magnetite and strontium hexaferrite is very attractive as they mimic the living tissue. In this context, Elhajjar et al. [45], presented a summary of the research conducted in the last two decades, related to the advances in magnetostrictive polymer composites (MPCs) and their applications. However, these are being limited by the complexity of this problem at numerous levels. In this context, the time, strain amplitude, frequency, magnetic field, shear rate, and temperature are expected to influence the forces ordering the particles and have not yet been studied systematically. Furthermore, a unified understanding of the interaction between the electromagnetic behavior, mechanical loads and thermal conditions is also needed. On the other hand, the internal cracking or interface degradation may lead to reduced cyclic magnetostriction as the strain transfer between particles and matrix within the composite is strongly reduced. Finally, the difficulty in conducting representative experiments is limiting the ability to develop advanced modeling approaches.

Recently, some of the authors [46] have found that in Fe75Ga25/silicone composites the Villari effect is due to the change of the cross section of the sample when it is submitted to a tensile stress and a model has been developed whose predictions fit very well with the experimental results. The origin of this effect is completely different from that of the classical Villari effect which, as it was mentioned previously, is due to the change of the magnetic permeability of the material; so this new effect could be better named “strain-Villari effect” to avoid any confusion with the classical Villari effect.

In this work it has also been obtained that in composites submitted to a magnetic field of 1 T after manufactured a good signal of the Villari effect is obtained without the necessity of applying a magnetic field to the sample during the measuring process. This result increases the interest in using these materials in strain and force sensors because the use of a primary coil is saved.

All these results bring out the relationship between the Villari effect and the mechanical properties of the composites. In order to further these results, in this article a study of the Villari effect in Fe81Ga19/silicone composites in function of the concentration and size of the soft magnetic particles has been developed. The Villari effect has also been studied in function of the magnetic state of the sample. The results reinforce the idea of the change of the cross section of the composite as the origin of the Villari signal in this kind of material.

## 2. Materials and Methods

Composites with CeysMs-Tech silicone matrix and Fe-Ga magnetic particles reinforcement have been manufactured. Fe-Ga particles have been chosen as a magnetic filler because they exhibit relatively high magnetostriction.

In order to obtain Fe-Ga particles high purity (99.9%) Fe and Ga metals were used as raw materials and its manufacture has been carried out in two steps.

First, alloys ingots of Fe81Ga19 were prepared by induction melting under vacuum atmosphere. From these ingots, ribbons of about 2 mm wide and 60 µm thick were produced by planar flow casting in vacuum atmosphere with a roll speed of 17.5 m/s.

Secondly, 25 g of manually cropped ribbons of Fe81Ga19 were milled under vacuum atmosphere using a planetary ball mill (Retsch, PM 100 model, TENCAM, Changsha, China). A 500 mL 1.2080 tool steel jar with 20 tempered steel balls of 10 mm in diameter were used as a milled container, setting a ball to powder ratio of 40:1 with a rotational speed of 400 rpm. To reduce further powder contamination, no lubrication or process control agents were added. The milling process lasted 2 h and it was interrupted every 20 min to dissipate the accumulated heat. In order to trace the evolution on the particle size, during each milling interruption, particles were sieved using A STM E-11/95 sieves forming 4 different groups of particle sizes: under 50 µm, 150–180 µm, 180–250 µm, and above 250 µm. Each group were weighted and carefully returned to the jar to restart the milling; this procedure was repeated until the 2 h of milling was reached.

Once the metallic reinforcements had been sieved and classified in different size distribution ranges, two types of polymer matrix composites materials were manufactured: 1) using different reinforcement contents and particles size <50 µm, and 2) varying the size distributions, leaving constant the Fe81Ga19 particles content (60 wt.%). Table 1 shows Fe81Ga19/Silicone composite materials analyzed in this work. In all cases the mixture of the metal reinforcements and the silicone matrix (Silicone Ceys Ms-Tech, L’Hospitalet de Llobregat, Spain) are carefully mixed for 5 min, using a stirrer bar at room temperature, guaranteeing a good homogenization of the distribution of the powders and avoiding degradation of the polymeric material. Next, the mixture was put into a cylindrical-shaped mold with a height of 50 mm, a diameter of 5 mm in the central part and 8 mm at the ends, to obtain the samples for the measurement of the Villari effect, and into a mold in the shape of a rectangular prism with a height of 50 mm and 25 mm^2^ of cross section to manufacture the samples for the measurement of Young’s modulus.

**Figure 1:**
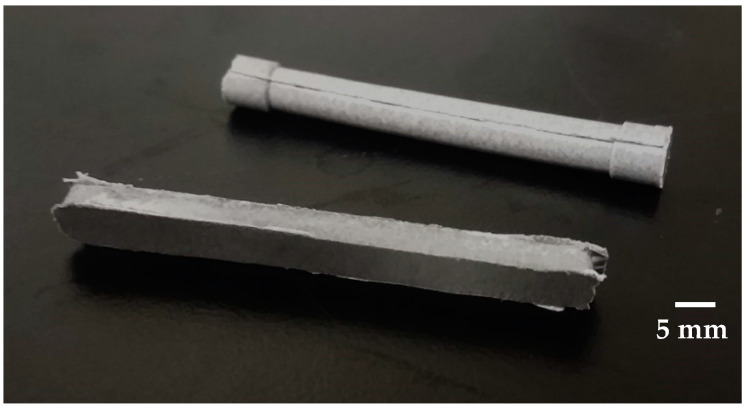
Picture of the composites: Cylindrical for Villari measurements and rectangular prims for measurements of microindentation.

Finally, the samples were cured for 36 h without applying any magnetic field (isotropic composites). After manufacturing, the isotropic composites were subjected to an applied magnetic field of 1 T in the longitudinal direction of the sample magnetizing it in this direction. A picture of the final shape of the composites is shown in Figure 1.

The mechanical properties of the composites were carried out by means of the Young’s modulus measurement. The Young’s modulus of the samples was measured by microindentation (P-h) curves. The measurements of microindentation were performed in a Microtest machine (MTR3/50-50/NI) using a Vickers indenter. The details of the method followed have been shown elsewhere [47].

**Figure 2:**
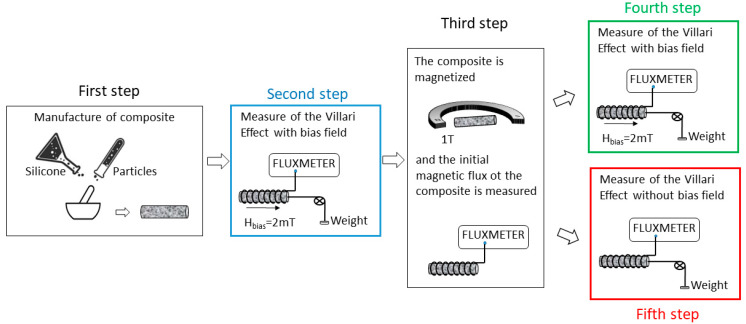
Scheme of Villari measurements.

In order to study the influence of the elastic and magnetic properties of composites on their Villari response, the measurements have been carried out in different steps, as shown in Figure 2.

The classical induction method was used to obtain the remnant magnetization of the composites after being submitted to an applied magnetic field of 1 T. The measurements were obtained by means of a fluxmeter integrator (Magnet-Physik EF 4, Köln, Germany) connected to a pickup coil that surrounds the composite. This coil of 7 mm inner diameter consists of 20,000 turns of copper wire of 0.05 mm diameter. The magnetic characterization of the Fe81Ga19 particles was carried out at room temperature using a vibrating sample magnetometer (EV9–VSM 2.2 T, MicroSense, Edinburgh, UK).

**Figure 3:**
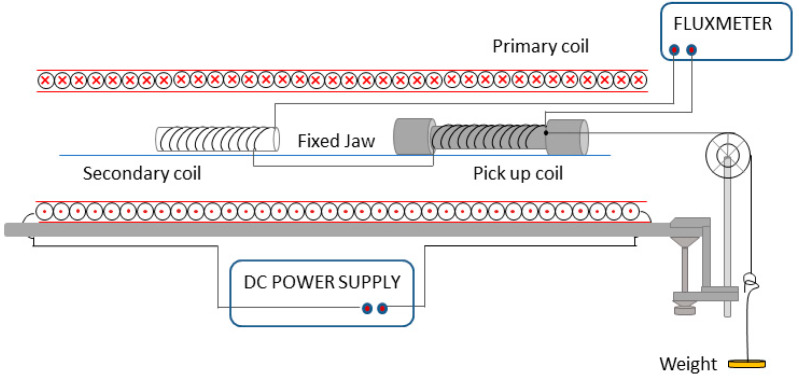
Experimental setup for measuring the Villari effect.

The experimental system to measure the Villari effect has been shown elsewhere [46], and basically consists of a direct power supply which provides an electrical current to a primary coil to magnetize the sample and a pick up coil that surrounds the sample. The Villari signal is obtained by means of a fluxmeter connected to the pickup coil that surrounds the sample, which is also connected to another identical secondary winding in series opposition. The pickup provides a signal proportional to B, and the secondary winding provides a signal proportional to μ_0_Ha. Therefore, the fluxmeter provides a signal proportional to M. The tensile stress was applied to the sample adding different weights to a holder fixed to one end of the sample while the other was attached to a fixed jaw. The scheme of the experimental set up is shown in Figure 3.

Five measurements in loading/unloading cycles of the Villari effect were performed to ensure the repeatability of the results. In the range of the measurements performed in our work there is a good repeatability of the results and no hysteresis effect have been observed. On the other hand, a good linearity is obtained in all the measurements. The lack of hysteresis and the linearity of the signal make this kind of material very promising for use in sensor technology, because these two characteristics are fundamental in the signal used in this field.

The measurements have been performed until a deformation of about 10% because higher stresses produce defects in the subjection parts of the samples.

## 3. Results and Discussion

**Figure 4:**
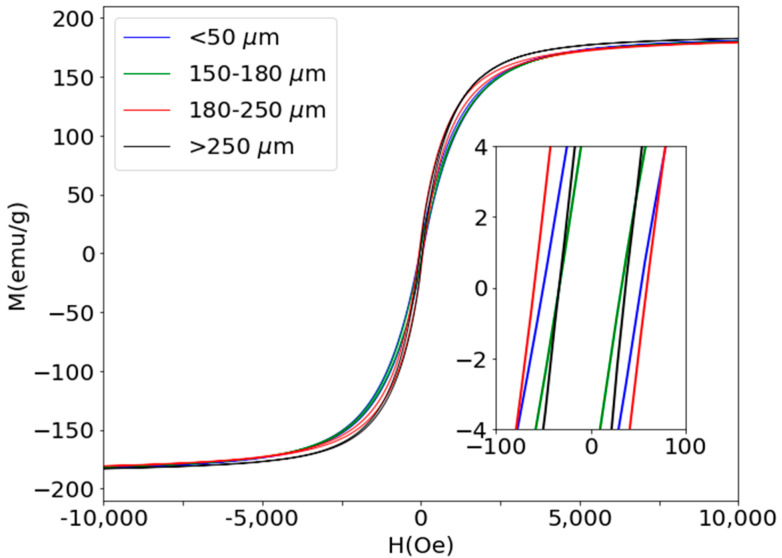
Hysteresis loops of the magnetic particles. The inset shows the enlargement of the central part of the loops.

The hysteresis loops and the magnetic properties of the different Fe81Ga19 magnetic particles used in this work are shown in Figure 4 and Table 2.

Although the results shown in Table 2 do not present a clear correlation between the size of the microparticles and the value of the coercive field, in all cases the soft magnetic behavior of these particles is confirmed.

**Figure 5:**
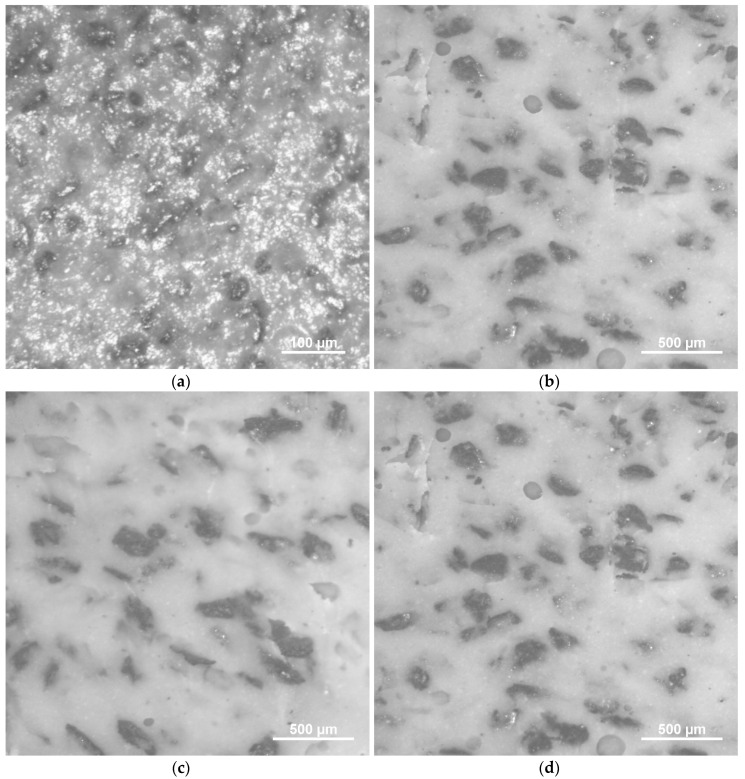
Optical micrographs showings microstructure of Fe81Ga19/silicone composites with particles size: (**a**) <50 µm and content of 45 wt.%; (**b**) 150–180 µm; (**c**) 180–250 µm; (**d**) > 250 µm and content of 60 wt.%.

Figure 5 shows the images of the composites make with the different sizes of particles used. The photographs show a certain dispersion in shape and size of the particles. The images also show a random distribution of the particles in all the composites, and therefore the shape anisotropy due to the non-rounded particles would be averaged, resulting in the isotropic composites. No aggregates are observed that could contribute to an increase in the coercive field of the composites [48]. The different distribution of sizes observed would explain the behavior of the coercive field.

### 3.1. Influence of the Content of Reinforcement

**Figure 6:**
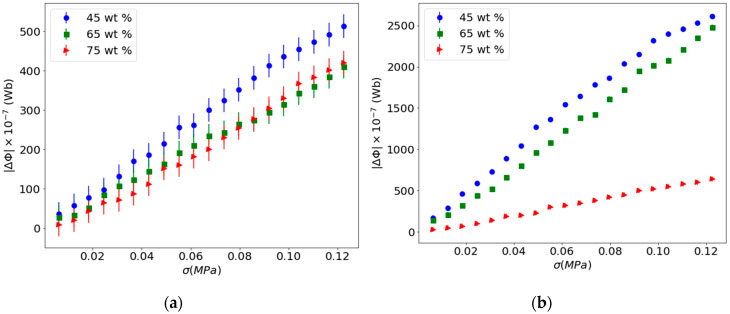
Villari effect in function of the filler concentration: (**a**) As-manufactured samples; (**b**) after being submitted to a magnetic field of 1 T, second and fourth steps of the procedure, respectively.

To study the influence of the reinforcement content in the Villari effect in this kind of composite, the concentrations of 45, 65 and 75 wt.% of particles with size <50 μm have been used. The Villari signal has been measured in as-manufactured samples and in samples submitted to a magnetic field of 1 T after cured as indicated in Figure 2 in the second and fourth steps. In these steps a bias magnetic field of 2 mT has been applied for the measurements of the Villari effect. In the last step it was not necessary to apply the bias field to obtain a Villari signal due to the remnant magnetization of the composites. The results obtained in the second and fourth steps of the procedure are shown in Figure 6.

Figure 6a shows few differences between the Villari signals obtained for the different manufactured composites. After being submitted to the magnetic field of 1 T, in Figure 6b it can be observed a dramatic increase in the Villari signal corresponding to the composites of 45 wt.% and 65 wt.%, while for the 75 wt.% a slight difference is observed.

**Figure 7:**
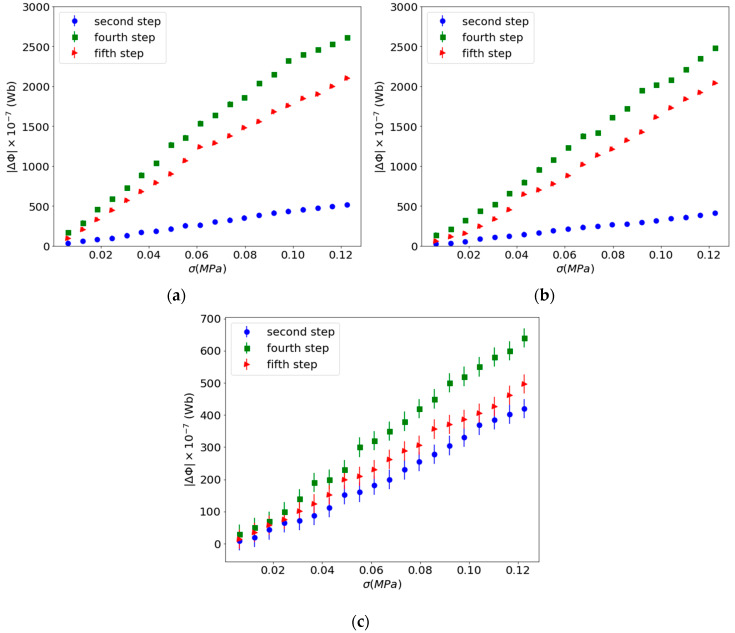
Results of the Villari effect in the different steps of the experiment: (**a**) Composites with 45 wt.%; (**b**) composites with 65 wt.%; (**c**) composites with 75 wt.%.

These results suggest that the initial magnetization of the composites after being subjected to the magnetic field of 1 T affects their Villari response. The applied magnetic field produces an alignment of the magnetization of the particles resulting in a net magnetization of the composite material that increases with increasing concentration of the particles, so the Villari signal should increase with the concentration of the fillers. However, as can be seen, the results show an opposite behavior; when the filler concentration increases, the Villari effect decreases. This is because in this kind of composite there is an important contribution to the Villari signal due to the reduction in the magnetic flux linked to the reduction in the cross section of the composites when submitted to a stress [46]. In this way, the increase in the reinforcement content produces a high rigidity of the composite that reduces the Villari effect because there is a smaller reduction in the surface of the composite with the stress. This interpretation also explain the increase in the Villari signal in the samples submitted to the field of 1 T because, in this case, the magnetization of the composite is higher, so an equal reduction in the cross section of the sample will produce a great decrease in the magnetic flux through it. To highlight the results Figure 7 shows the signals of the Villari effect measured in function of the different steps of the procedure.

In general, our results are consistent with those presented by other authors who have studied the behavior of ferrogels. Filipcsei et al. [43], detected a weak effect of magnetodeformation for gels contained randomly distributed magnetic particles, as well as, no magnetodeformation and swelling was observed for gels containing aligned particles in the polymer matrix with the application of low magnetic fields (300 mT). On the other hand, Zubarev et al. [42], indicate that if the particle concentration is small and they are randomly distributed in the matrix, the sample can deform in the magnetic field direction (depending on particles shape). While, a composite with a high concentration of the particles can only elongate due to effect of the particles spatial disposition (short ranged order). Therefore, to estimate amplitude of this magnetodeformation, one should consider both mechanical (due to the local deformations of the matrix) and magnetic interactions between the particles.

Furthermore, different behavior can be observed in the Villari signals measured in the second and fourth steps depending on the concentration of reinforcement. The 45 wt.% and 65 wt.% composites show a considerable increase in the Villari effect when subjected to the 1 T magnetic field. For the 75 wt.% composite, there are slight differences between that submitted to the 1 T magnetic field and the as-manufactured one. These results reinforce the idea of the relationship between the Villari signal and the elastic properties of the composite.

Figure 7 also shows that these composites present a Villari signal without applying a bias field during the measurement (fifth step).

To verify the relationship between the Villari effect and the mechanical properties, the Young’s modulus of the composites has been obtained by microindentation (P-h curves). In a previous work [47], some of the authors have adapted the Oliver and Pharr [49,50,51] method to the kind of composites used in this work. In this method, the hardness is obtained from the loading-unloading curves by the expression:

(1) $$H=\frac{P_{max}}{A}$$

where *P_max_* is the maximum load and *A* is the contact area.

The effective elastic modulus *E_eff_* was calculated by the expression:

(2) $$E_{eff}=\frac{S}{\beta \frac{2}{\sqrt{\pi }}\sqrt{A}}$$

where *S* is the slope of unloading P-h curves and *β* is a correction factor dependent of the indenter.

Finally, the elastic modulus was calculated from *E_eff_* considering the elastic modulus of the indenter *E_i_*, the Poisson’s ratio of the indenter *υ_i_* and that of the silicone *υ*, by the expression:

(3) $$E=\frac{\left(1-\upsilon^{2}\right)}{\frac{1}{E_{eef}}-\frac{\left(1-\upsilon_{i}^{2}\right)}{E_{i}}}$$

**Figure 8:**
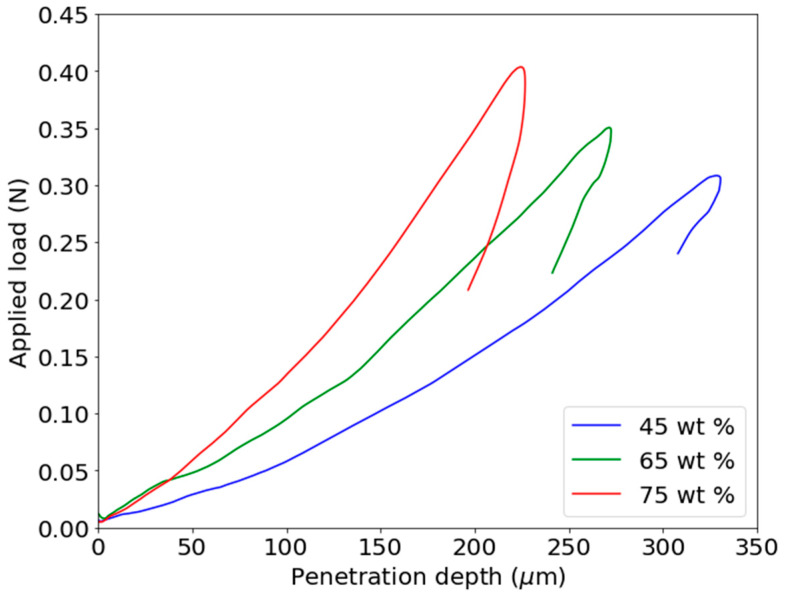
P-h curves of the composites with 45, 65 and 75 wt.% of Fe81Ga19.

In Figure 8, the P-h loading and unloading curves of the composites filled with 45, 65 and 75 wt.% of magnetic particles are shown.

From these curves the Young’s modulus shown in Table 3 are obtained.

**Figure 9:**
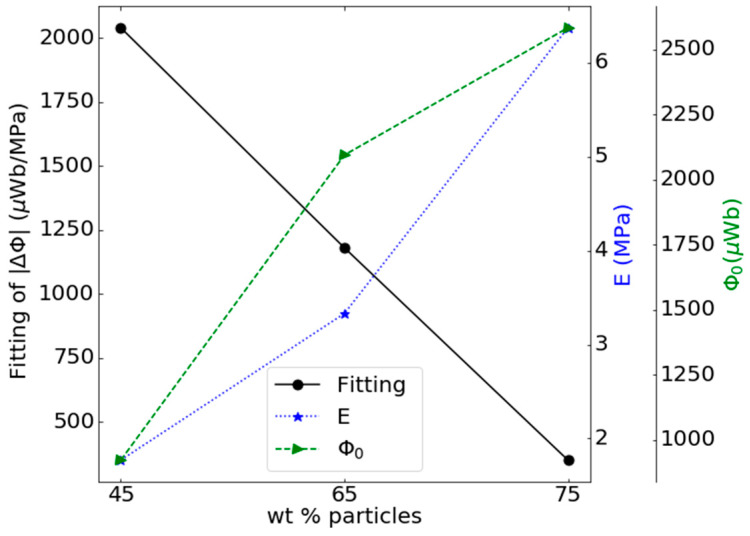
Slope of the fitting of the Villari signal, Young’s modulus and initial magnetic flux of the composites in function of the reinforcement content for the fifth step of the procedure.

The Villari signals of Figure 6 have been adjusted to a quadratic function in order to compare the slopes obtained from the adjustments with the mechanical and magnetic properties of the composites. In this way, Figure 9 shows the slope of the adjustment of the Villari signals, the Young’s modulus and the initial magnetic flux of the samples in function of the concentration of the particles in the composite.

The results of Figure 9 show that increasing Young’s modulus of composites decreases the slope of the fitting of the Villari signal. This result corroborates the influence of the mechanical properties on the Villari effect in this kind of material. This can be explained because as the Young’s modulus of the composites increases, the reduction in the cross section of the sample when under tension is smaller and, therefore, the slope of the Villari effect should be smaller.

Figure 9 also shows that the slope of the adjustment in the Villari signal decreases with increasing initial magnetization. This result contradicts the fact that an increase in the initial magnetization of the sample should contribute to an increase in the measured Villari signal. This indicates a predominant influence of the mechanical properties on the Villari effect in these composites and that the origin of the effect is different from that of the classical Villari effect so, to highlight its origin and to avoid any confusion with the classical Villari effect we will call him strain-Villari effect.

### 3.2. Influence of the Reinforcement Size

The influence of the size of the magnetic particles of the composites in the strain-Villari effect has also been studied. For this samples with a concentration of 60 wt.% and sizes of 150–180 μm, 180–250 and >250 μm have been used.

**Figure 10:**
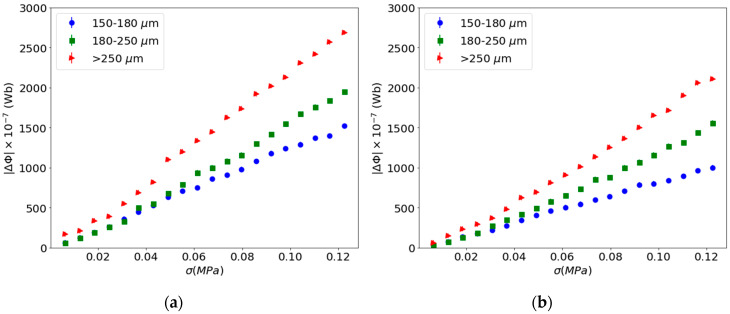
Strain-Villari effect of the composite submitted to a magnetic field of 1 T: (**a**) Measured with field of 2 mT (fourth step); (**b**) measured without field (fifth step).

The strain-Villari effect has been measured in composites submitted to a magnetic field of 1 T after cured, both with and without applying a magnetic field of 2 mT during the measurement process, fourth and fifth steps of the procedure, respectively. Figure 10 shows the results obtained.

From this figure, for the same concentration of the reinforcement, with increasing particle size the Villari signal increases. Other authors have discussed the importance of magnetic history for the detection process and demonstrated the importance of remnant magnetization in the case of the gels with large magnetic particles [44].

The Young’s modulus of these composites had been measured in a previous work [47] by microindendation and the results obtained are indicated in Table 4.

**Figure 11:**
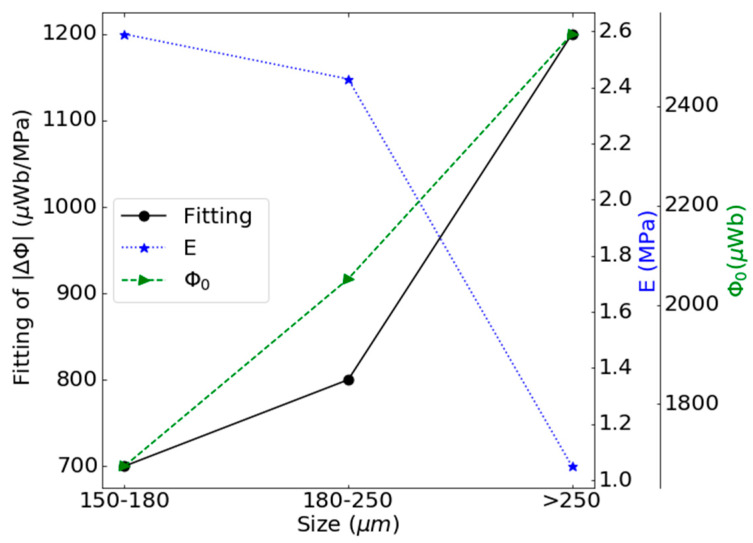
Slope of the fitting of the strain-Villari signal, the Young’s modulus of the composites and the initial magnetic flux of the composites in function of the particle size for the fifth step of the procedure.

As in the previous section, the Villari signals from these samples have again been adjusted using a quadratic function and the results of the adjustment slope together with Young’s modulus and the initial magnetic flux are shown in Figure 11.

In this case, the increase in the size of the filler particles produces a decrease in the Young’s modulus and an increase in the initial magnetic flux of the composites, which favors the increase in the strain-Villari effect, as shown in the Figure 11.

To explain the relationship between the Villari effect with the mechanical properties and the appearance on the effect without the necessity of applying a magnetic field during the measuring process, a simple model was previously developed by some of the authors [46]. In this model, it is assumed that due to the narrow range of applied tensile stress the volume of the sample keeps constant, but due to the low Young’s modulus the cross-section changes when submitted to stress so.

(4) $$\left(l+\Delta l\right)S^{\prime }=\mathrm{lS}$$

where l is the initial length of the sample and Δl the length variation after deformation; S and S’ its initial and final, respectively, cross section.

Considering the relationship between the stress σ, strain ε and Young’s modulus E, the cross section of the stressed sample is:

(5) $$S^{\prime }=\frac{\mathrm{SE}}{E+\sigma }$$

According to Brown [52] when the composite is submitted to stress there is also a change in its magnetization:

(6) $$\Delta M={\beta \sigma }^{2}$$

being β a constant.

From these effects, the change in the magnetic flux will be:

(7) $$\Delta \phi =\mu_{0}N\left(M^{\prime }S^{\prime }-\mathrm{MS}\right)$$

In our case the stress applied is very low and the variation of the magnetization could be negligible; however, the change of the cross section of the composite will produce a change in the magnetic flux:

(8) $$\phi =\mu_{0}\mathrm{NM}\left(S^{\prime }-S\right)$$

Variation in the cross section of a composite when stress is applied decreases with increasing Young’s modulus. Therefore, according to Equation (8), the variation of the magnetic flux through the sample will decrease, according with the results obtained in this paper.

## 4. Conclusions

The Villari effect has been studied in composites formed by an elastomer matrix with a low Young’s modulus and soft magnetic particles. The results have shown the existence of a strong relationship between the Villari response and the mechanical properties of the samples. These properties play the most important role in the Villari effect. In this way, it has been verified that when the Young’s modulus decreases, the Villari signal increases.

The increase in the Villari response of the samples due to their initial magnetization depends strongly on the elastic properties of the composite. In this sense, in composites with a reinforcement concentration of 45 and 65 wt.%, the magnetic state of the composite plays an important role in the Villari signal, increasing when the magnetization of the sample is greater. However, the effect of the initial magnetization is smaller in composites with 75 wt.%, due to its elastic properties.

These results confirm that the reduction in the cross section of the sample when subjected to stress is the main origin of the Villari effect in this kind of composite. This fact means that the origin of this effect was completely different from that of the classical Villari effect, and could be better named as the “strain-Villari effect” to highlight its origin and to avoid any confusion with the classical Villari effect.

## Figures and Tables

**Table 1:** Parameters of manufactured composites.

	Particles Size (µm)	Weight Fraction (wt.%)
Influence of reinforcement content	<50	45
		65
		75
Influence of particles size	150–180	60
	180–250	
	>250	

**Table 2:** Magnetic properties of the particles in function of the size.

Size of the Particles (µm)	Hc (Oe)	Mr (emu/g)	Ms (emu/g)
<50	52 ± 1	9 ± 1	183 ± 1
150–180	32 ± 1	6 ± 1	183 ± 1
180–250	58 ± 1	13 ± 1	182 ± 1
>250	33 ± 1	10 ± 1	184 ± 1

**Table 3:** Young’s modulus of the composites with 45, 65 and 75 wt.% of Fe81Ga19.

Weight Fraction (wt.%)	Young’s Modulus (MPa)
45	1.77 ± 0.03
65	3.33 ± 0.03
75	6.37 ± 0.03

**Table 4:** Young’s modulus of the composites in function of the magnetic particle size.

Particle Size (µm)	Young’s Modulus (MPa)
150–180	2.59 ± 0.05
180–250	2.43 ± 0.04
>250	1.05 ± 0.02
